# CD44 Suppression Improved the Chemosensitivity of HT-29 Colorectal Cancer Cells to 5-Fluorouracil and Inhibited Cell Migration

**DOI:** 10.34172/apb.2023.053

**Published:** 2022-07-02

**Authors:** Souzan Najafi, Zohreh Rahimi, Behzad Mansoori, Ali Mohammadi, Fatemeh Mohammadnejad, Mohammad Amini, Ahad Mokhtazadeh, Zahra Asadzadeh, William Chi-Shing Cho, Behzad Baradaran

**Affiliations:** ^1^Student Research Committee, Faculty of Medicine, Kermanshah University of Medical Sciences, Kermanshah, Iran.; ^2^Immunology Research Center, Tabriz University of Medical Sciences, Tabriz, Iran.; ^3^Medical Biology Research Center, Kermanshah University of Medical Sciences, Kermanshah, Iran.; ^4^Department of Clinical Biochemistry, Faculty of Medicine, Kermanshah University of Medical Sciences, Kermanshah, Iran.; ^5^Department of Cancer and Inflammation Research, Institute for Molecular Medicine, University of Southern Denmark, Odense, Denmark.; ^6^Student Research Committee, Tabriz University of Medical Sciences, Tabriz, Iran.; ^7^Department of Clinical Oncology, Queen Elizabeth Hospital, Hong Kong SAR, China.

**Keywords:** CD44, 5-Fluorouracil, Colorectal cancer, Chemosensitivity, Cell migration

## Abstract

**Purpose:**

CD44 plays a pivotal role through tumorigenesis by regulating cancer cell metastasis, stemness, and chemosensitivity and is considered a promising therapeutic target for human cancers, including colorectal cancer (CRC). Therefore, the present research aimed to examine the simultaneous therapeutic effect of CD44 silencing and 5-fluorouracil (5-FU) on *in vitro* tumorigenesis of CRC cells.

**Methods:**

CD44 expression was initially evaluated in TCGA datasets and CRC tissues. Furthermore, functional analysis was performed on HT-29 CRC cells overexpressing CD44. The cells were transfected with CD44 siRNA and then treated with 5-FU. Consequently, to explore the combination therapy effect on cell viability, migration, apoptosis, and chromatin fragmentation, we performed MTT assay, scratch assay, Annexin V/PI staining and DAPI staining assays, respectively. The spheroid and colony formation assays were further employed to investigate stemness features. The gene expression at protein and mRNA levels were explored using western blotting and qPCR.

**Results:**

Our findings illustrated that CD44 was significantly overexpressed in CRC tissues compared to normal samples. The suppression of CD44 considerably promoted the chemosensitivity of HT-29 cells to 5-FU by apoptosis induction. Also, the combination therapy led to overexpression of apoptotic genes, including P53, caspase-3, and caspase-9, as well as downregulation of AKT1 expression. Furthermore, CD44 suppression, separately or combined with 5-FU, hindered stemness properties in HT-29 cells via downregulation of Sox2 and Nanog expression. Besides, the combination therapy remarkably downregulated MMPs and suppressed CRC cell migration.

**Conclusion:**

Considering its involvement in chemosensitivity to 5-FU, CD44 could be suggested as a potential target for improving the efficiency of CRC chemotherapy.

## Introduction

 As one of the lethal human malignancies, colorectal cancer (CRC) is the third most widespread cancer worldwide.^1,2^ The high mortality rate of CRC patients is mostly due to the failure of conventional treatment methods, as the consequence of distant metastasis and unresponsive to chemotherapy, and insensitivity to immune therapy.^3^ As a result, patients’ five-year survival expectancy is poor and around 12% in advanced stages.^4^ Approximately fifty percent of CRC patients suffer from metastasis after resection of primary tumors.^3^ Furthermore, acquired and intrinsic drug resistance was reported to limit the patient’s response to CRC treatment; as an example, only 10–15% of advanced CRC patients were reported to effectively respond to 5-fluorouracil (5-FU) as a single chemotherapy agent.^5^ Meanwhile, identifying molecular mechanisms participating in colorectal tumorigenesis through modulating cell metastasis and chemosensitivity could be an effective way to develop new therapeutic approaches for this malignancy.

 Nowadays, multiple chemotherapeutic drugs are used for CRC treatment, including 5-FU. This antimetabolite chemotherapeutic agent is considered the first-choice chemotherapy in the treatment of CRC. 5-FU drug, through interfering with the synthesis of DNA and generating fractures in its strands, leads to damage to genomic structure and suppression of cancer cell proliferation.^6^ However, the appearance of resistance in most patients is considered an essential obstacle to successful treatment of CRC using 5-FU chemotherapy,^7^ demanding new strategies to overcome molecular mechanisms decreasing CRC cell chemosensitivity and to improve the efficiency of CRC treatment using 5-FU chemotherapeutic agent.

 The cluster of differentiation 44 (CD44), a single-pass transmembrane protein, is transcribed from a highly conserved gene located on chromosome 11 in the human genome.^8^ CD44 is expressed virtually in all types of human cells,^9^ and through binding to different ligands, including hyaluronic acid, activates molecular signaling pathways participating in the regulation of cell adhesion, migration, proliferation, and differentiation.^10^ The high expression of CD44 has also been reported to interrupt multiple cellular and molecular processes. It is involved in the progression of human malignancies, as diverse as, chronic lymphocytic leukemia, breast cancer, gastric adenocarcinoma, and bladder cancer.^11-14^ Notably, the previous studies have clarified that CD44 overexpression correlates with tumorigenesis and the metastasis in CRC patients^15^ and plays an imperative role in migration of CRC cells through regulating AKT phosphorylation.^16^ Besides, CD44 suppression using small interfering RNA (siRNA) was shown to inhibit tumor growth in CRC mouse models.^17^ Moreover, CD44 was illustrated to be involved in tumor cell chemosensitivity in acute myeloid leukemia, non-small cell lung cancer and breast cancer^18-21^; then, it suggests that CD44 play an essential role in the regulation of cancer cell chemo-responsiveness.

 Therefore, considering that overcoming mechanisms by which tumor cells resist chemotherapies could improve the survival rate of CRC patients, the current research was designed to explore the *in vitro* effects of CD44 silencing using specific siRNAs, as a targeted therapy strategy, combined with 5-FU drug on human CRC cells. Subsequently, the effect of combination therapy, as a promising treatment strategy, was also investigated on CRC cell migration and stemness.

## Materials and Methods

###  In-silico investigation of CD44 expression through colorectal tumorigenesis

 To initially investigate if CD44 exhibits aberrant regulation through colorectal tumorigenesis, its expression status was evaluated using the online public datasets in The Cancer Genome Atlas (TCGA). For this aim, CD44 expression data for 635 colorectal cases and 51 normal cases were retrieved using the Xena Functional Genomics Explorer (https://xenabrowser.net/) and then analyzed.

###  Preparation of CRC tissue samples 

 Fresh-frozen CRC and adjacent (marginal) normal tissue specimens of 10 patients diagnosed with primary CRC were collected from the Emam Reza hospital (Tabriz, Iran) during surgical resection. The marginal samples were at least 6 cm away from the primary tumor site. Before RNA extraction, all samples were kept in liquid nitrogen. All patients were given written informed consent.

###  Cell culture

 SW480, HT-29, LS180, and HCT116 CRC cell lines were purchased from Institute Pasture Cell Bank (Tehran, Iran). The cells were cultivated in RPMI-1640 medium (Sigma, USA) containing 10% FBS (Gibco, USA) and 1% penicillin/streptomycin (Invitrogen; Thermo Fisher Scientific, Inc., USA) and incubated at 37°C and atmosphere providing 95% humidity and 5% CO_2_. The cultivated cells were trypsinized (Trypsin-EDTA 0.25%, Gibco, USA) and sub-cultured as they reached 70-80% confluency.

###  siRNA transfection 

 Regarding that HT-29 cells express high levels of CD44 compared to other cell lines, they were selected for subsequent experiments. Briefly, the cells at the density of 2 × 10^5^ cells/well were seeded into 6-well plates and cultivated for 24 hours to achieve 70-80% confluency. Afterward, using in-vitro jetPEI^TM^ (Polyplus transfection, Germany) and regarding supplied procedures, the cells were transfected with siRNAs targeting CD44 mRNA (Santa Cruz Biotechnology, California, USA) (Table 1) and scramble siRNA as the negative control in various amounts, including 40, 60 and 80 pmol. The transfected cells were incubated for 6 hours, and then the medium was changed with a complete RPMI medium. Subsequently, after 24-72 hours of further cultivation, total RNA was extracted, and CD44 expression levels were evaluated using qRT-PCR to determine transfection efficiency.

**Table 1:** CD44 siRNA sequences

**SiRNA**	**Sequences (5’-3’)**	
CD44 (1)	Sense	UUUUGGAAAUCACUAAUAGtt
	Antisense	CUAUUAGUGAUUUCCAAAAtt
CD44 (2)	Sense	AAUGCAAACUGCAAGAAUCtt
	Antisense	GAUUCUUGCAGUUUGCAUUtt
CD44 (3)	Sense	AAGAGAAAGGAAGUUUUUCtt
	Antisense	GAAAAACUUCCUUUCUCUUtt

###  RNA extraction and qRT -PCR 

 To determine the gene expression at mRNA levels, total RNA was isolated from tissue samples and treated cells using the GeneAll RiboEX reagent (Biotechnology, South Korea) regarding the manufacturer’s protocols. According to the absorbance ratio at wavelengths of 260 nm and 280 nm, RNA quality and concentration were evaluated using the ThermoFisher Nanodrop spectrophotometer (Scientific Life Sciences, USA). Then, to synthesize complementary DNA (cDNA) from mRNAs, one µg of extracted RNA was reverse transcribed using the BIOFACT cDNA synthesis kit (Daejeon, South Korea), regarding supplied protocols. Subsequently, the evaluation of changes in gene expression was done using the SYBR Premix Ex Taq (Takara Bio, Japan) in the Roche Diagnostics LightCycler^®^ 96 system (Mannheim, Germany). The small subunit 18S rRNA (18S) gene was used as the reference gene for normalization. Table 2 summarizes the sequences of used primers.

**Table 2:** The primer sequences

**Target name**	**F/R**	**Sequences (5’ to 3’)**
18 s	F	GATCAGATACCGTCGTAGTTCC
	R	CTGTCAATCCTGTCCGTGTC
CD44	F	CAAGCCACTCCAGGACAAGG
	R	ATCCAAGTGAGGGACTACAACAG
MMP2	F	CCCACTGCGGTTTTCTCGAAT
	R	CAAAGGGGTATCCATCGCCAT
MMP3	F	CAAAGGATACAACAGGGACCA
	R	ATCTTGAGACAGGCGGAACC
MMP9	F	TTGACAGCGACAAGAAGTGG
	R	GCCATTCACGTCGTCCTTAT
AKT	F	GCTGCACAAACGAGGGGAG
	R	CCGCTCCGTCTTCATCAGCT
P53	F	AAAGTCTAGAGCCACCGTCC
	R	AATCCAGGGAAGCGTGTCA
Nanog	F	TGTCTTCTGCTGAGATGCCT
	R	TTTCTTGACCGGGACCTTGT
Sox2	F	ACATGTGAGGGCCGGACAGC
	R	TTGCGTGAGTGTGGATGGGATTGG
caspase-3	F	ATGGTTTGAGCCTGAGCAGA
	R	CATCCACACATACCAGTGCGTA
caspase-8	F	CTGGTCTGAAGGCTGGTTGTT
	R	GTGACCAACTCAAGGGCTCAG
caspase-9	F	GCAGGCTCTGGATCTCGGC
	R	GCTGCTTGCCTGTTAGTTCGC

###  Western blotting

 Protein extraction from treatment groups was carried out using the Santa Cruz RIPA lysis buffer according to the manufacturer’s procedures. The extracted cellular proteins (25 μg) were separated using SDS polyacrylamide gel electrophoresis and transferred to a polyvinylidene difluoride membrane (Roche Diagnostics GmbH, Rotkreuz, Switzerland) by semidry immunoblotting. Using 0.5% Tween-20 solution (solved in PBS), the membrane was blocked for 1.5 hours in a shaking condition at 37°C. Then, the membrane was exposed to specific monoclonal antibodies targeting CD44 and beta-actin, as the internal control (Santa Cruz Biotechnology), overnight at 4℃. After washing the membrane, anti‐mouse secondary antibodies (Santa Cruz Biotechnology) were added, and the membrane was incubated for 50 minutes at room temperature. Finally, the protein bands were visualized via the imaging system of western blotting (Sabz Biomedicals, Iran).

###  MTT assay

 Briefly, HT-29 cells, at the density of 1.5 × 10^4^ cells per well, were seeded into 96‐ well plates and cultivated for 24 hours. Subsequently, the cells were exposed to 5-FU in various concentrations (5- 100 μg/mL) and incubated for a further 24 hours. Then, MTT solution (5 mg/mL; Sigma‐Aldrich) was added to wells, and the cells were maintained in a dark condition for 4 hours at 37°C. For dissolving the formazan crystals, dimethyl sulfoxide (DMSO, 200 µL per well) was replaced with the medium, and the plate was shaken for 10 minutes. The absorbances at the wavelength of 570 nm were explored via a microplate reader (Sunrise^TM^, Tecan). Furthermore, to evaluate the CD44 suppression effect on 5-FU chemosensitivity, HT-29 cells were pre-treated with CD44 siRNA and incubated for 24 hours. Then, they were treated with the aforesaid concentrations of 5-FU, and after 24 hours were subjected to MTT assay.

###  Apoptosis analysis (annexin V/PI staining)

 HT-29 cells (2.5 × 10⁵ cells per well) were cultivated in a 6-well culture plate. The cells were transfected with CD44 siRNA, then exposed to 5-FU, and cultured for 48 hours. Afterward, the cells were harvested and washed with PBS. Then, the cells were stained using the BD Biosciences Annexin V/PI kit regarding the manufacturer’s procedures. The portion of viable, necrotic, and apoptotic cells was then determined using the MACSQuant flow‐cytometry. To analyze the obtained data, FlowJo version 7.6 software (TreeStar Inc., USA) was employed.

###  Nuclear staining (DAPI)

 To investigate the chromatin fragmentation status in treatment groups, DAPI staining was employed. HT-29 cells were seeded at a density of 1.5 × 10^4^ cells per well into a 96-well plate. Then, the cells were transfected with CD44 siRNA and treated with 5-FU, separately or simultaneously. After 48 hours, the cultured cells were incubated with 4% paraformaldehyde for 1 hour to get fixed. To permeabilize the cells, 0.1% Triton-X-100 diluted in PBS was added to the wells, and the plate was kept in the incubator for 15 minutes. The cells were then stained with 0.1% DAPI solution in the darkness for 10 minutes. Finally, chromatin fragmentation status in treated and untreated cells was explored using the Cytation^TM^ 5 Cell Imaging Multi-Mode Reader (BioTek, USA).

###  Cell migration assay

 To understand if CD44 knockdown combined with 5-FU could influence HT-29 cell mobility, a wound-healing (scratch) assay was carried out. In a 24-well plate, the cells, at a density of 3 × 10^5^ cells per well, were seeded to reach 70-80% confluency. Then, using a yellow sterile pipette tip, a wound area (scratch) was created on monolayer cells. Afterward, the cells were treated with CD44 siRNA, 5-FU, and CD44 siRNA/5-FU. Then, the migration of cells from edges to gap area was photographed and analyzed at different hours, including 0, 24, and 48 hours after treatments.

###  Colony formation assay

 To investigate the capability of a single HT-29 cell to grow and form a colony, the cells (1 × 10³ cells per well) were seeded into a 6-well plate. The cells after transfection with CD44 siRNA and treatment with 5-FU were incubated for two weeks until visible clones formed. The formed cellular colonies were incubated with 0.5% crystal violet (Sigma, USA). The related images to each treatment group were taken using an inverted light microscope.

###  Spheroid formation assay

 The simultaneous effects of CD44 suppression and 5-FU treatment on HT-29 cell stemness were investigated using the spheroid formation assay. In a 6-well plate, at a density of 2.5 × 10^5^ cells per well, the cells were cultured for 24 hours. The transfected cells with or without 5-FU treatment were separated by trypsin, and the cells (5 × 10^3^ cells per well) were reseeded into a 96-well culture plate. The cells were incubated in the 2x RPMI-1640 medium containing 10 % Matrigel (Corning Matrigel Basement Membrane Matrix, Life Sciences, USA)and 10% FBS for 10 days. In the final step, an inverted microscope was used to take images of the formed spheroid in each group.

###  Statistical analysis

 All data are shown as the mean ± standard deviation (SD) of triplicated experiments. The statistical differences between treatment groups were investigated via Student’s t‐test and one-way analysis of variance using GraphPad Prism 6 (San Diego, CA, USA). It was considered statistically significant when the obtained *P* values were less than 0.05.

## Results and Discussion

 Due to distance metastasis and resistance to developed therapeutic strategies, including chemotherapy and immune therapy, CRC is known as one of the deadliest malignancies worldwide, particularly in advanced stages^3,22^; demanding novel therapeutic approaches for better management of patients. Recent studies have established that CD44 oncogene plays a significant role in the initiation and progression of various human cancers through modulating multiple processes, including cancer cell chemosensitivity and metastasis.^23,24^ In particular, this putative cancer stem cells marker was shown to be overexpressed through CRC tumorigenesis and its increased expression levels were associated with lymph node and distant metastasis,^25^ indicating its therapeutic value for CRC. Subsequently, the present research aimed to examine the therapeutic effects of CD44 knockdown using specific siRNA on metastatic features of CRC cells and their sensitivity to 5-FU chemotherapy, as a promising therapeutic method.

###  CD44 overexpression in CRC tissues and cell lines

**Figure 1:**
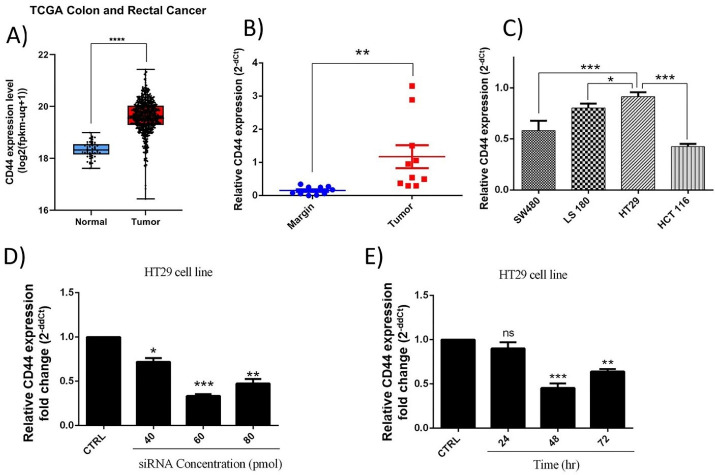
CD44 expression levels. **(A)** CD44 upregulation in CRC samples retrieved from TCGA CRC dataset; ****P < 0.0001. **(B)** CD44 expression was explored in colorectal cancer tissues using qRT-PCR; n = 10; the data demonstrated overexpression of the mRNA levels of CD44 in tumoral samples compared to healthy tissues; **P < 0.01. **(C)** CD44 mRNA levels in CRC cell lines; HT-29 cells showed the highest levels CD44mRNA expression. *P < 0.05; ***P < 0.001. **(D) **CD44 mRNA expression in transfected cells with siRNA at concentrations of 40, 60, and 80 pmol. **(E)** The effect of transfection of CD44 siRNA (60 pmol) on its expression through different incubation times, including 24, 48 and 72 h. *P < 0.05; **P < 0.01; ***P < 0.001; ns = non-significant

 The obtained results from TCGA colon and rectal datasets illustrated that CD44 shows significantly (*P* < 0.0001) higher expression levels in CRC samples compared to normal specimens (Figure 1A). Besides, to confirm CD44 dysregulation through colorectal progression, its expression levels were evaluated in CRC tissues (n = 10) compared to adjacent normal colorectal tissues using qRT-PCR. Our results demonstrated that CD44 is significantly (*P* < 0.01) upregulated at mRNA levels compared to adjacent non-cancerous tissues (Figure 1B). These results were consistent with previous studies showing CD44 upregulation through colorectal tumorigenesis.^25^

 Furthermore, evaluation of CD44 expression in CRC cell lines illustrated that CD44 is highly expressed in these cells. However, highly metastatic HT-29 cells showed more increased levels of CD44 expression compared to SW-480 (*P* < 0.001), LS180 (*P* < 0.05), HCT-116 (*P* < 0.001) cell lines (Figure 1C). Then, considering the obtained results, it was suggested that the malignant features of HT-29 cells might be more influenced by CD44 expression compared to three other cell lines. So, to better follow the effects of CD44 suppression, HT-29 cell line was selected for further investigations.

###  CD44 expression was suppressed using specific siRNAs

 HT-29 cells were transfected with CD44 siRNA at different amounts, including 40, 60, and 80 picomole for 24, 48, and 72 hours to efficiently suppress CD44 expression. As shown in Figure 1D-E, the obtained results illustrated that 60 pm of siRNA was adequate to effectively decrease the expression of CD44 until 48 hours in HT-29 cells. Therefore, further experiments were done in the same conditions.

###  CD44 suppression improved in vitro cytotoxicity 5-FU 

**Figure 2:**
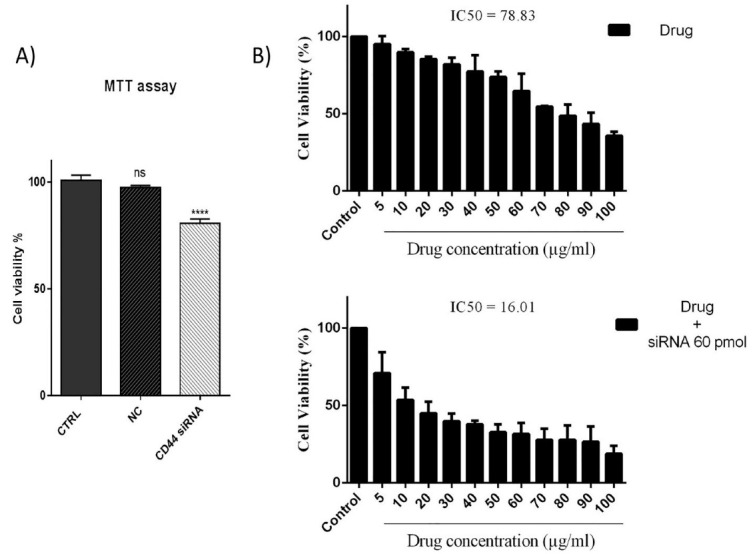
MTT assay results **(A)** CD44 suppression significantly reduced HT-29 cell viability. ****P< 0.0001; ns = non-significant.** (B)** CD44 silencing increases the sensitivity of HT-29 cells to 5-FU treatment in a dose‐dependent way

 To demonstrate the effect of CD44 suppression on the viability and 5-FU chemosensitivity of HT-29 cells, MTT assays was done. As illustrated in Figure 2A, despite not observing a significant difference between the control and scramble group (NC), CD44 suppression led to a considerable decrease in the survival rate of HT-29 cells. Furthermore, the obtained results (Figure 2B) indicated that the combined treatment with CD44 siRNA (60 pmol) and 5-FU (0–100 μg/mL) suppressed the viability of HT-29 cells more efficiently than 5-FU treatment alone. In fact, pretreatment of cells with CD44 siRNA could reduce the IC50 value of 5-FU from 78.83 μg/mL to 16.01 μg/mL; it was evidenced that CD44 may participate in the regulation of CRC cell responsiveness to 5-FU treatment.

###  CD44 suppression is independent of 5-FU therapy in HT-29 cells

**Figure 3:**
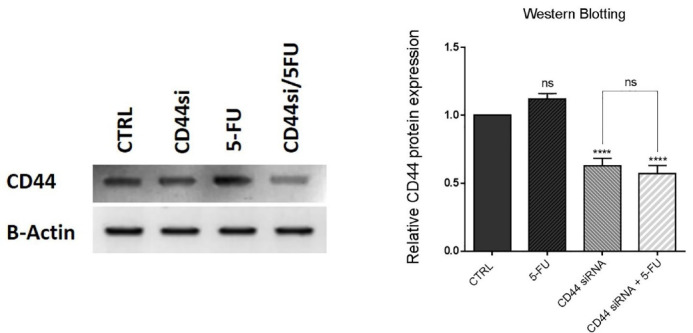
Western blotting results. The knockdown of CD44 is independent of 5-FU therapy. CD44 protein expression levels decreased through CD44 suppression in CD44 siRNA and combination groups. However, exposure to 5-FU treatment hadn’t significant effect on CD44 protein expression; ****P< 0.0001, ns = non-significant

 To investigate that CD44 suppression effects aren’t influenced by 5-FU treatment, western blotting was carried out to assess the expression of CD44 at protein levels in HT-29 cells through combination therapy with CD44 siRNA and 5-FU. Our results indicated that CD44 suppression led to a significant (*P* < 0.0001) reduction in its protein expression levels compared to the control. Besides the ineffectiveness of 5-FU treatment on CD44 expression, no significant difference was seen in CD44 protein expression between the cells transfected with CD44 siRNA alone and the cells simultaneously transfected with CD44 siRNA and treated with 5-FU (Figure 3). The findings showed that CD44 silencing using siRNA would exert its effects independent of the effect of 5-FU therapy on the expression of CD44 in HT-29 cells.

###  CD44 suppression increased apoptosis induction by 5-FU in HT-29 cells

**Figure 4:**
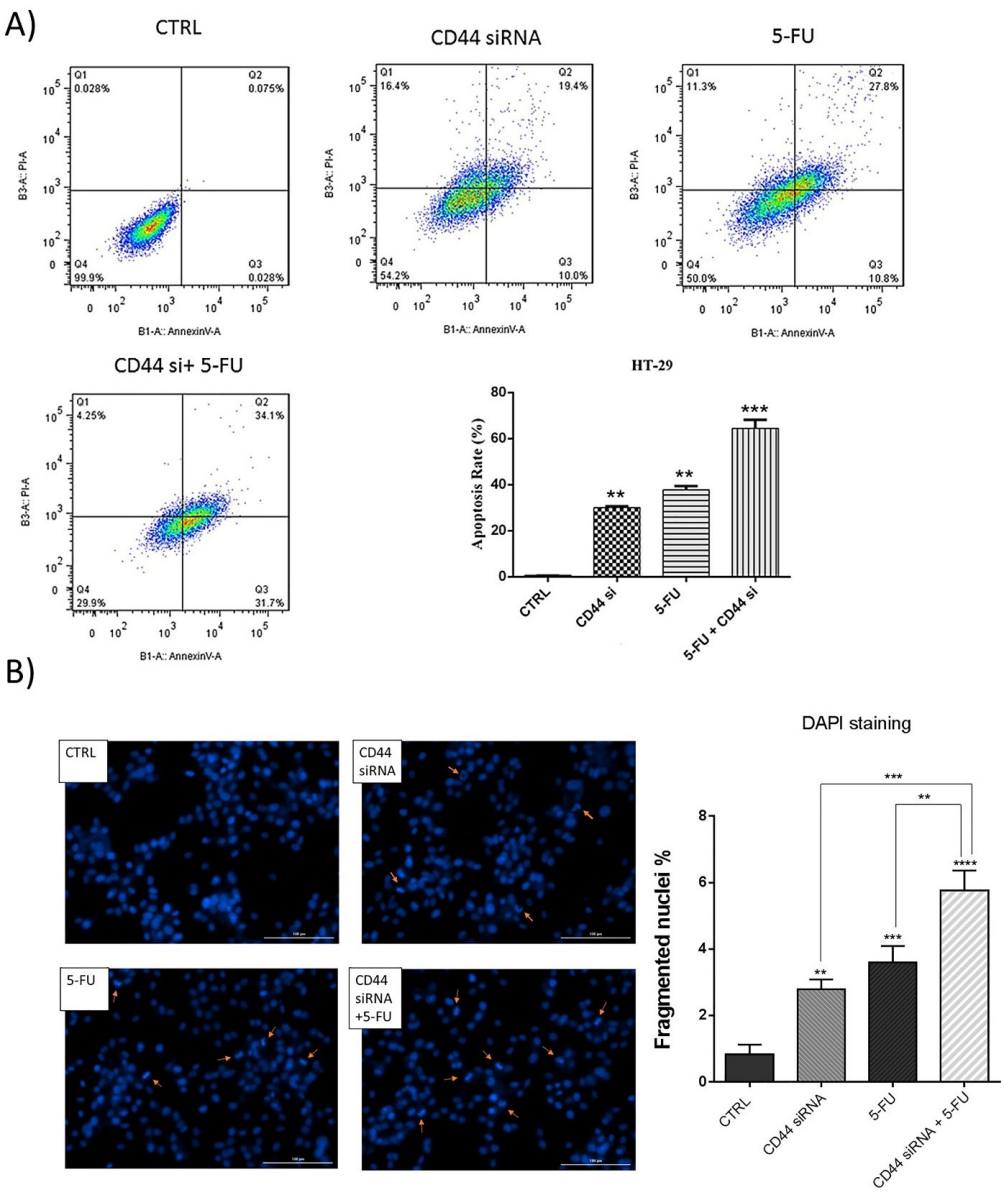
Apoptosis assay. **(A) **Flow cytometry analysis byannexin V-FITC/PI staining demonstrated that CD44 knockdown and treatment with 5-FU cooperatively increased the percentage of early and late apoptotic cells. **(B)** DAPI staining further validated the apoptosis induction in each treatment group. The results showed an increase in fragmented nuclei rate in combination groups compared to separate treatment with CD44 siRNA and 5-FU; **P < 0.01, ***P < 0.001, ****P < 0.0001

 Because the combined CD44 siRNA and 5-FU significantly decreased cell viability, to investigate underlying mechanisms, we evaluated apoptosis induction through treatment of HT-29 cells with these agents using Annexin V-FITC/PI staining. The obtained results evidenced that suppressing CD44 expression and 5-FU treatment could significantly provoke apoptosis in HT-29 cells. Transfection of cells with CD44 siRNA raised the percentage of early apoptotic cells (V-FITC ^+^ / PI - ) and late apoptotic cells (V-FITC ^+^ / PI ^+^ ) to 10% and 19.4%, respectively. Also, the early and late apoptotic cell percentage increased to 10.8% and 27.8% after treatment of HT-29 cells with 5-FU. However, a notable rise was observed in the percentage of apoptotic cells (65.8%) by simultaneous use of CD44 siRNA and 5-FU compared to separate treatments (Figure 4A). In fact, the simultaneous suppression of CD44 and exposure to 5-FU increased the rate of V-FITC ^+^ /PI - and V-FITC ^+^ /PI ^+^ cells to 31.7% and 34.1%. Besides, DAPI staining confirmed the apoptosis induction in these cells. The results showed that, compared to the control (0.83%), the number of cells with fragmented nuclei in CD44 siRNA transfected (2.78%, *P* < 0.01) and 5-FU treated (3.59%, *P* < 0.001) groups was considerably higher. However, the percentage of fragmented nuclei was higher in the combination group (5.75%, *P* < 0.0001) compared to 5-FU (*P* < 0.001) and CD44 siRNA (*P* < 0.01) groups (Figure 4B); suggesting that CD44 suppression increases apoptosis induction when it is simultaneously used with 5-FU.

**Figure 5:**
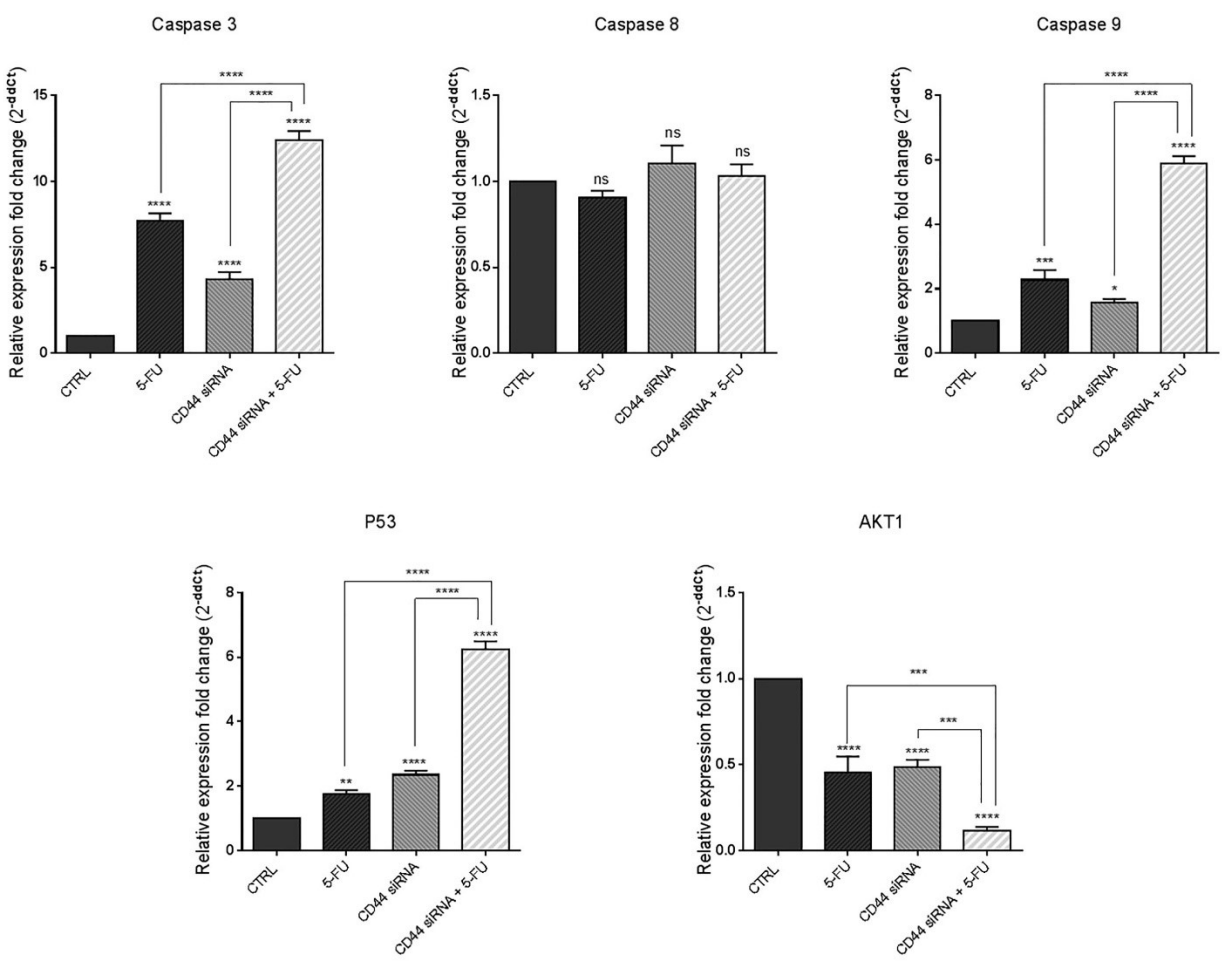
Caspase 3, caspase 8, caspase 9, P53, and AKT1 mRNA expression in treatment groups. The combination therapy caused the significant upregulation of proapoptotic genes and downregulation of pro-survival/anti-apoptotic genes; *P < 0.05, **P < 0.01, ***P < 0.001 and ****P < 0.0001, ns = non-significant

 To further clarify the molecular mechanism underlying apoptosis induction through CD44 knockdown, qRT-PCR was used to quantify the changes in the expression of major apoptosis and cell survival regulators. As shown in Figure 5, compared to the controls, suppression of CD44 expression and treatment with 5-FU simultaneously led to significant (*P* < 0.0001) increase in the mRNA levels of caspase 3. Also, despite that no significant change in caspase 8 levels was observed, CD44 siRNA and 5-FU treatment increased the expression levels of caspase 9 in HT-29 cells (*P* < 0.05 and *P* < 0.001, respectively); however, compared to separate treatment with CD44 siRNA and 5-FU, caspase 9 expression was higher in the combination group (*P* < 0.0001). Furthermore, p53 mRNA was also significantly overexpressed through suppressing CD44 expression and exposure of cells to 5-FU chemotherapy (*P* < 0.0001 and *P* < 0.01, respectively). The highest level of p53 expression was observed in the combination group. Besides, it was evidenced that combined suppression of CD44 expression and 5-FU treatment could cooperatively downregulate AKT compared to control (*P* < 0.0001). Collectively, these results revealed CD44 suppression, either alone combined or with 5-FU chemotherapy, could provoke cell death through upregulation of intrinsic apoptotic pathway and modulating the expression of survival and apoptosis-related genes.

 Lakshman et al previously reported that overexpression of CD44 in SW620 CRC cells could induce resistance to apoptosis via changes in caspase-3, caspase-9, Bcl-xl, and Bak expression and modulating the mitochondrial pathway.^26^ Nizar m. Mhaidat et al also showed that induction of apoptosis by 5-FU was dependent on caspase-9 activity in CRC.^27^ Besides, CD44 was reported to be involved in regulating the chemosensitivity in other human cancers. It was illustrated that CD44 knockdown could enhance the sensitivity of chemoresistant non-small-cell lung carcinoma cells to Cisplatin by overexpression of Bax/Bcl-2 ratio and activation of caspase-3.^21^ Also, CD44 silencing was shown to improve the responsiveness of hepatocellular carcinoma cells to Doxorubicin, and to induce apoptosis in these cells.^24^

 Furthermore, Herishanu et al reported that high expression of CD44 could activate MAPK/ERK and PI3K/AKT signaling and has an anti-apoptotic effect in chronic lymphocytic leukemia cells.^28^ Dhar et al also reported that CD44 is required for AKT activation, which resulted in the blocking of the p53 genomic surveillance response.^29^ Li et al reported that the combined Kaempferol and 5-FU significantly deactivated PI3K/AKT signaling and increased apoptosis in CRC cells.^30^ Then, it could be implied that CD44 suppression combined with 5-FU treatment might modulate CRC cell viability through downregulation of AKT signaling.

###  CD44 knockdown in combination with 5-FU inhibited HT-29 cell migration 

**Figure 6:**
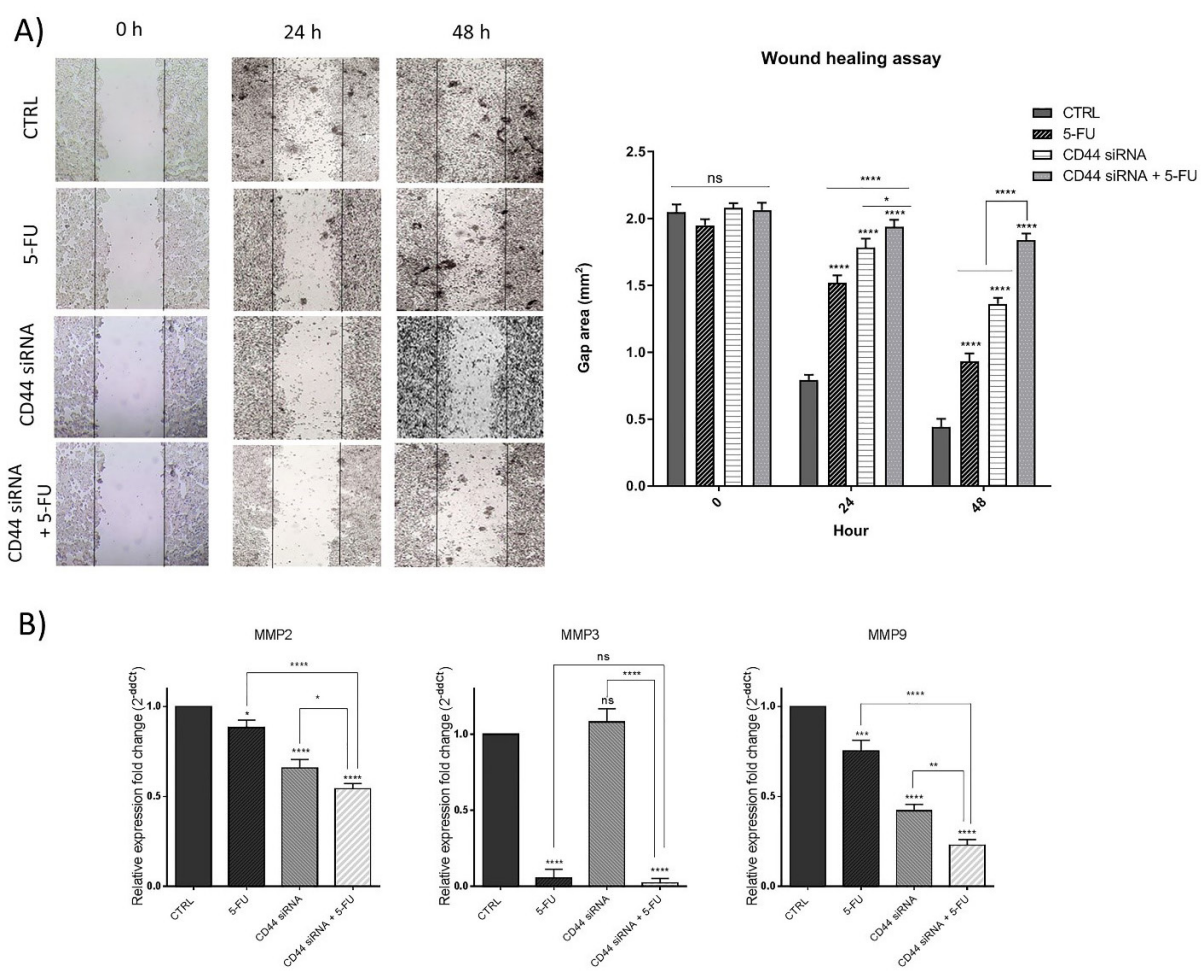
Scratch assay results. **(A)** The results indicated that CD44 suppression and 5-FU cooperatively could reduce HT-29 cell mobility. **(B)** The mRNA expression levels of MMPs, including MMP2, MMP3, and MMP9 in treatment groups; *P < 0.05; **P < 0.01; ***P < 0.001; ****P < 0.0001

 To explore the simultaneous effects of suppressing CD44 and 5-FU treatment on the motility of HT-29 cells, a wound-healing (scratch) assay was done. Our findings indicated that CD44 knockdown and 5-FU treatment separately led to a decrease in the migration ability of HT-29 cells compared to the control group, 24 hours and 48 hours (*P* < 0.01 and *P* < 0.001) after treatments. However, as illustrated in Figure 6A, in the combination group, the number of cells that migrated to the gap area was more remarkably decreased than separate treatments, demonstrating the anti-migratory ability of combination therapy on CRC cells. Considering the notable reduction of migrated cells, we explored the expression level of MMPs as the main modulators of cancer cell migration and metastasis using qRT-PCR. The results indicated that suppression of CD44 expression (*P* < 0.0001) and exposure to 5-FU (*P* < 0.05) separately could decrease the expression levels of MMP2 compared to control (Figure 6B). The lowest levels of MMP2 expression were achieved through combination therapy (*P* < 0.0001). Also, it was illustrated that despite no significant change through CD44 suppression, 5-FU treatment also substantially (*P* < 0.0001) reduced MMP3 mRNA levels in 5-FU and combination groups (*P* < 0.0001) compared to control. Besides, CD44 siRNA and 5-FU treatment cooperatively downregulated MMP9 expression, as another metastasis marker, compared to control (*P* < 0.0001).

 In agreement with the results obtained, Lee and colleagues previously reported that CD44 shRNA-mediated suppression was capable of inducing apoptosis in HCT-116 colon cancer cells and significantly inhibited cell migration and invasion.^31^ The matrix metalloproteinases are overexpressed through colorectal tumorigenesis, showing a correlation with advanced stages malignancy and patients’ poor prognosis.^32^ Consistently, it was evidenced that there is a notable correlation between CD44 expression and MMP2/9 in cancer models that suggests the inhibition of the CD44-MMPs axis as a promising therapeutic target for suppressing metastasis.^33^ Then, in the present study, we also evidenced that MMPs suppression mediated by CD44 knockdown combined with 5-FU treatment may be considered a promising way to improve patient outcomes in advanced stages of CRC.

###  Co-treatment of CD44 suppression and 5-FU inhibited the colony and sphere formation 

**Figure 7:**
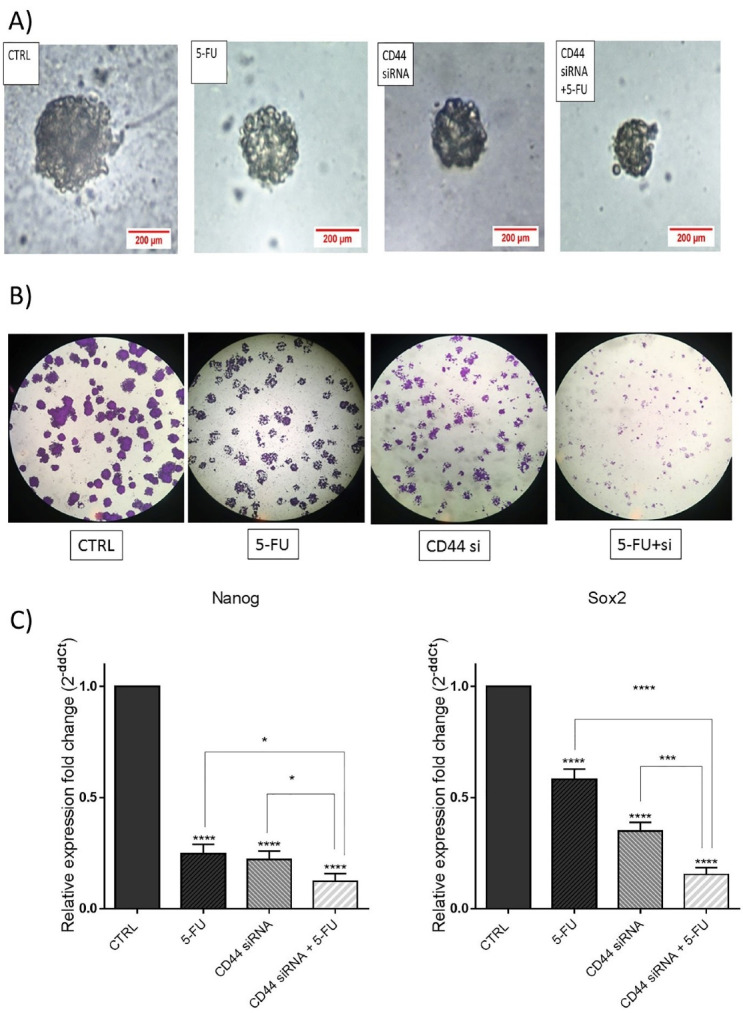
The combination of CD44 siRNA and 5-FU reduced stemness properties in HT-29 cells. **(A) **Spheroid formation assay was performed. Data indicated that single groups had a larger size of spheroids than the combination CD44 siRNA/5-FU group. **(B)** The colony formation assay demonstrated the smaller size of formed colonies in the combination group compared to the control and separate treatments. **(C)** mRNA expression levels of Nanog and Sox2 after transfection of CD44 siRNA with or without 5-FU were evaluated via qRT‐PCR. Data represent the means ± SD of experiments. *P < 0.05; ***P < 0.001; ****P< 0.0001

 In the current study, the combined effects of suppressing CD44 and 5-FU treatment on inhibition of HT-29 cell stemness were also explored by colony and sphere formation assays. The results demonstrated that CD44 knockdown and 5-FU treatment alone remarkably reduced the ability of HT-29 cells to form spheroids compared to the control. As seen in Figure 7A, the size of spheroids was reduced through the combination therapy more than separate treatments. Furthermore, HT-29 cells clonogenic ability were decreased through suppressing CD44 and 5-FU treatment. As depicted in Figure 7B, a notable decrease was observed in the size of formed colonies in separately treated cells compared to control. However, the combination therapy suppressed the colony formation and proliferation ability of these cells more effectively than separate treatments.

 To clarify underlying molecular mechanisms, Sox2 and Nanog mRNA expression levels, as important stemness markers, were also measured via qRT-PCR. The results indicated that the expression levels of these genes were notably reduced through transfection of cells with CD44 siRNA separately or combined with 5-FU compared to the control groups (Figure 7C). Therefore, it was suggested that CD44 suppression combined with 5-FU could effectively suppress the stemness of CRC cells through modulating its major regulators.

 Accordingly, Lee et al reported that CD44 knockdown exhibited a significant diminution in colony formation ability of HCT116 colon cancer cells.^31^ Also, CD44 suppression was reported to hamper A549 lung cancer cell proliferation and colony formation.^34^ Besides, Lee et al demonstrated that 5-FU treatment in combination with melatonin was able, by downregulation of cellular prion protein, to restrain colon cancer stem cells and subsequent inhibition of stemness markers, including Nanog, Oct4, ALDH1A1 and Sox2.^35^

## Conclusion

 In brief, the obtained results from the present research signified that CD44 knockdown could effectively diminish the proliferation of HT-29 cells and improved their chemosensitivity to 5-FU treatment via enhancement of apoptosis induction, illustrating CD44 involvement in the CRC cell responsiveness to 5-FU treatment. Besides, considering that CD44 suppression and 5-FU treatment cooperatively inhibited CRC cell *in vitro* growth and migration, their combination could be regarded as a beneficial strategy to develop novel approaches for the improvement of CRC chemotherapy. However, there is a need for more *in vivo* and *in vitro* studies to further validate the value of this combination therapy for CRC treatment.

## Competing Interests

 All authors declare that they have no conflict of interest.

## Ethical Approval

 This study was approved by the Ethics committee of Tabriz University of Medical. Sciences, Tabriz, Iran (Code: IR.TBZMED.REC.1399.293).
